# Comparing structured versus unstructured suicide risk assessment across health professionals and the general population: a vignette-based study protocol (CARE)

**DOI:** 10.3389/fpsyt.2026.1773604

**Published:** 2026-03-30

**Authors:** Gabriele Torino, Giuseppe Delvecchio, Raffaella Calati, Sara Magliocca, Chiara Moltrasio, Nicholas Citerà, Letizia Toracca, Paolo Brambilla

**Affiliations:** 1 Department of Pathophysiology and Transplantation, University of Milan, Milan, Italy; 2 Department of Neurosciences and Mental Health, Fondazione IRCCS Ca’ Granda Ospedale Maggiore Policlinico, Milan, Italy; 3 Department of Psychology, University of Milan-Bicocca, Milan, Italy

**Keywords:** health professionals, inter-rater reliability, suicide, suicide risk assessment, vignette study

## Abstract

**Background:**

Suicide risk assessment is a complex clinical task characterized by substantial variability in judgment and low predictive accuracy, even among experienced clinicians. Standardized instruments such as the Columbia Suicide Severity Rating Scale (C-SSRS) have improved the structure of assessments, yet inter-rater agreement remains limited, particularly for intermediate risk categories. Moreover, insufficient suicide assessment training among healthcare professionals and widespread misconceptions about suicide in the general population further compromise the accuracy and consistency of risk estimates. The CARE study aims to address these gaps by examining variability in suicide risk estimates and evaluating whether brief suicide assessment training and the use of the C-SSRS can enhance agreement across different groups and over time.

**Method:**

The study employs a vignette-based randomized experimental design to directly compare suicide risk estimates across ten participant groups, including mental health professionals, other healthcare workers, university students, and individuals without clinical backgrounds. At baseline, participants will be randomly assigned to an experimental condition involving brief training and the use of the C-SSRS or to a non-experimental condition requiring risk estimates based solely on subjective impressions and/or clinical experience. Each participant will evaluate six vignettes representing low, moderate, and high suicide risk. After two weeks, participants will complete a follow-up assessment with six additional vignettes to examine within-person consistency. Primary outcomes include between-group and within-subject discrepancies in risk estimates, while secondary outcomes focus on differences between C-SSRS and non-C-SSRS conditions.

**Discussion:**

The CARE study will provide one of the most comprehensive vignette-based evaluations of suicide risk assessment across healthcare professionals and laypeople. Findings may inform targeted training, support the integration of standardized tools, and guide public health strategies aimed at improving early recognition and consistent evaluation of suicide risk.

## 1 Introduction

Suicide represents a global burden (1), and its risk assessment is among the most complex challenges in the mental health field (2). Despite the critical importance of accurately identifying individuals at risk, studies have documented substantial variability in clinical judgments, even among experienced professionals (3, 4). Furthermore, given the difficulties in identifying algorithms or scales with high predictive value (5), partly due to low base rates and small samples in intervention studies (6), estimates based on clinical or personal experience play a critical role in suicide risk management even today. For instance, research conducted by Regehr et al. (7) revealed that factors such as age, stress levels, and scores on standardized questionnaires were among the factors most strongly associated with risk estimations in a sample of social workers and university students.

In light of these challenges, in recent years, there has been an increasing adoption of standardized suicide risk assessment instruments designed for use by clinicians and the general population. Among these, for example, the Ask Suicide-Screening Questions (ASQ) (8) is a brief screening tool composed of four ‘yes’ or ‘no’ items designed to identify suicidal ideation and past suicide attempts in non-clinical settings. Although the ASQ is primarily a screening instrument rather than a comprehensive risk assessment tool, it demonstrates how structured approaches to suicide-related questioning have been extended beyond specialist clinical contexts. It can be administered in under one minute by lay personnel and shows high sensitivity in different settings for detecting individuals who require further expert evaluation (9–11); however, it is intended for screening and referral purposes only and does not provide an assessment of suicide risk severity or imminence. Moreover, the Oxford Mental Illness Suicide tool (OxMIS) (12) is an innovative web-based tool that provides a percentage risk score of the likelihood of suicide in the months following the assessment. The OxMIS tool demonstrated encouraging results in terms of its predictive value and clinical feasibility; however, further external validation studies are necessary to substantiate its accuracy (13). Additionally, its intended utilization is restricted only to individuals diagnosed with severe mental illnesses (bipolar disorder or schizophrenia spectrum disorders), and its purpose is to serve as an adjunct to clinical decision-making, thereby positioning it for application by clinicians within a clinical context. Another example is represented by the Columbia Suicide Severity Rating Scale (C-SSRS) (14), which does not focus on specific subgroups of people and, importantly, is designed to assess suicide-related outcomes both for clinicians and for large-scale use in the general population. This factor, together with its easy-to-use design, has contributed to making the scale widely adopted worldwide and employed in suicide risk assessment in both clinical and non-clinical contexts. More recently, its official smartphone application has also been released (https://apps.apple.com/us/app/columbia-protocol/id1450966911), allowing everyone to answer the C-SSRS items and obtain an immediate estimate of suicide risk. Importantly, the C-SSRS is not intended as a predictive algorithm but as a structured tool to identify suicide-related warning signs and connect to aid resources, a function that remains relevant regardless of the broader debate on categorical risk stratification. Studies revealed high internal consistency and good discriminative ability in both clinical and research settings (14), as well as high predictive validity across diverse study samples (15–17). In 2012, it was endorsed by the Food and Drug Administration as the preferred instrument for measuring suicidal thoughts and behaviors in clinical trials, but it has also been subject to criticism and skepticism regarding its operational definitions of suicide-related outcomes and its psychometric properties (18).

Despite the current efforts to integrate sophisticated computational approaches to enhance the detection of suicidal behaviors (19), the degree of agreement of suicide risk assessment among both clinical and non-clinical raters remains limited, particularly for intermediate risk categories. As an example, a recent vignette-based experimental study found that interrater agreement in suicide risk classification was low among both trained psychotherapists and psychology students (20). The same study also showed that within-subject agreement was moderate for psychotherapists and low for psychology students, suggesting an association between clinical experience and consistency in subjective evaluations. In addition, as expected, the categories ‘low risk’ and ‘high risk’ yielded higher agreement rates compared to the ‘moderate risk’ one, highlighting a particularly critical gray area. Overall, previous articles emphasized that even trained clinicians performed only slightly better than chance when predicting suicide risk, and people without mental health or clinical training tended to perform worse or at best similarly to untrained professionals (21–23). To our knowledge, no peer-reviewed studies have directly compared the suicide risk assessment performed by licensed mental health professionals, residents in psychiatry, and psychology students with that of the general population and other healthcare professionals. Comparative research across these groups may provide valuable insights into the role of specialized training and the usefulness of standardized tools, such as the C-SSRS, in reducing variability across raters, ultimately enhancing clinical decision-making (24, 25). Indeed, given the low inter-rater reliability observed in suicide risk estimates even among mental health professionals (26), the use of standardized instruments designed for public use may represent a valuable resource for improving accuracy and consistency in risk evaluation among both clinicians and laypeople.

Building upon these premises, the protocol of the CARE (“Comparing structured versus unstructured suicide risk assessment across health professionals and the general population”) study described here seeks to fill these gaps in the literature to compare clinical suicide risk assessment performed by health professionals with suicide risk estimation provided by psychiatry residents, psychology students, and members of the general population, by using a vignette-based experimental design that includes a condition with and without a brief training on the use of the C-SSRS scale. In this context, participants from the general population are not intended to perform a clinical evaluation but rather to provide a judgment-based estimation of suicide risk, reflecting how individuals may interpret warning signs in everyday situations outside healthcare settings. Given the variability and low inter-rater reliability in clinical suicide risk estimation, employing a vignette-based design with follow-up consistency in risk estimation will enable a controlled assessment of consistency that would not be achievable in naturalistic clinical settings (27). Notably, the present study does not aim to validate suicide risk prediction models, but rather to examine how different individuals interpret and estimate suicide risk when exposed to the same information, a process that underlies both clinical formulation and everyday decision-making.

### 1.1 Aims and hypotheses

This study aims to compare suicide risk estimates across different populations and conditions. Specifically, the objectives are: (i) to compare suicide risk evaluations across the different study groups; (ii) to examine the temporal stability of suicide risk estimates when participants evaluate different but comparable clinical scenarios over time; and (iii) to assess whether the use of a structured instrument (C-SSRS) is associated with greater convergence in risk classification compared to unstructured judgment based on personal or clinical impressions. In addition to group-based comparisons, the study will examine whether differences are better explained by dimensional indicators of clinical experience rather than by professional labels alone.

Additionally, based on the expected results, the following hypotheses were formulated:

*HP 1*: Greater clinical experience and prior exposure to suicide-specific training will be associated with higher agreement and more consistent suicide risk estimates, regardless of professional category (Aim 1).

*HP 2*: Differences in risk estimation are expected to reflect variability in clinical exposure to acute suicidal presentations rather than disciplinary background per se (Aim 1).

*HP 3*: All study groups will show lower agreement for intermediate risk categories compared to low and high-risk categories (Aim 1 and 2).

*HP 4*: Greater within-subject variability is expected among individuals with limited or no clinical exposure to suicidal behavior compared to those with greater prior clinical exposure. This expectation is based on the idea that personal experiences, momentary emotional states, and situational cues may exert a stronger influence on individuals who are not familiar with clinical decision-making, whereas healthcare professionals are trained to rely primarily on clinical information and data (Aim 2).

*HP 5*: Participants assigned to the experimental condition (brief training on suicide risk assessment and use of the C-SSRS) will exhibit greater between-subjects and within-subject agreement in suicide risk estimates across all study groups (Aim 3).

## 2 Methods

### 2.1 Study design

The CARE study will employ a vignette-based randomized experimental design with experimental (C-SSRS) and non-experimental conditions (No-C-SSRS), which will focus on a brief suicidology training and the use of the C-SSRS scale for performing suicide risk estimates. This brief training module is intended to standardize the comprehension of the structured tool rather than to deliver skills-based training. The online survey questionnaire will be conducted through the Qualtrics software platform (https://www.qualtrics.com). Notably, the 18 vignettes that will be used in the present project were provided by the corresponding author of a previous study (20), who granted permission for their use in this project. These vignettes were translated into Italian specifically for the purposes of this study by members of the research team fluent in both languages and familiar with suicidology terminology. The translated version was then reviewed collectively by all authors to ensure semantic accuracy, conceptual equivalence, and clarity of clinical meaning within the Italian sociocultural context. Any discrepancies were resolved through consensus discussion, and the final version was approved by the full author group. No formal pilot testing was conducted before study launch, as the vignettes were originally developed for research purposes and are used here to examine comparative judgment patterns rather than to validate the scenarios themselves.

### 2.2 Population

#### 2.2.1 Selection of participants

**Figure 1:**
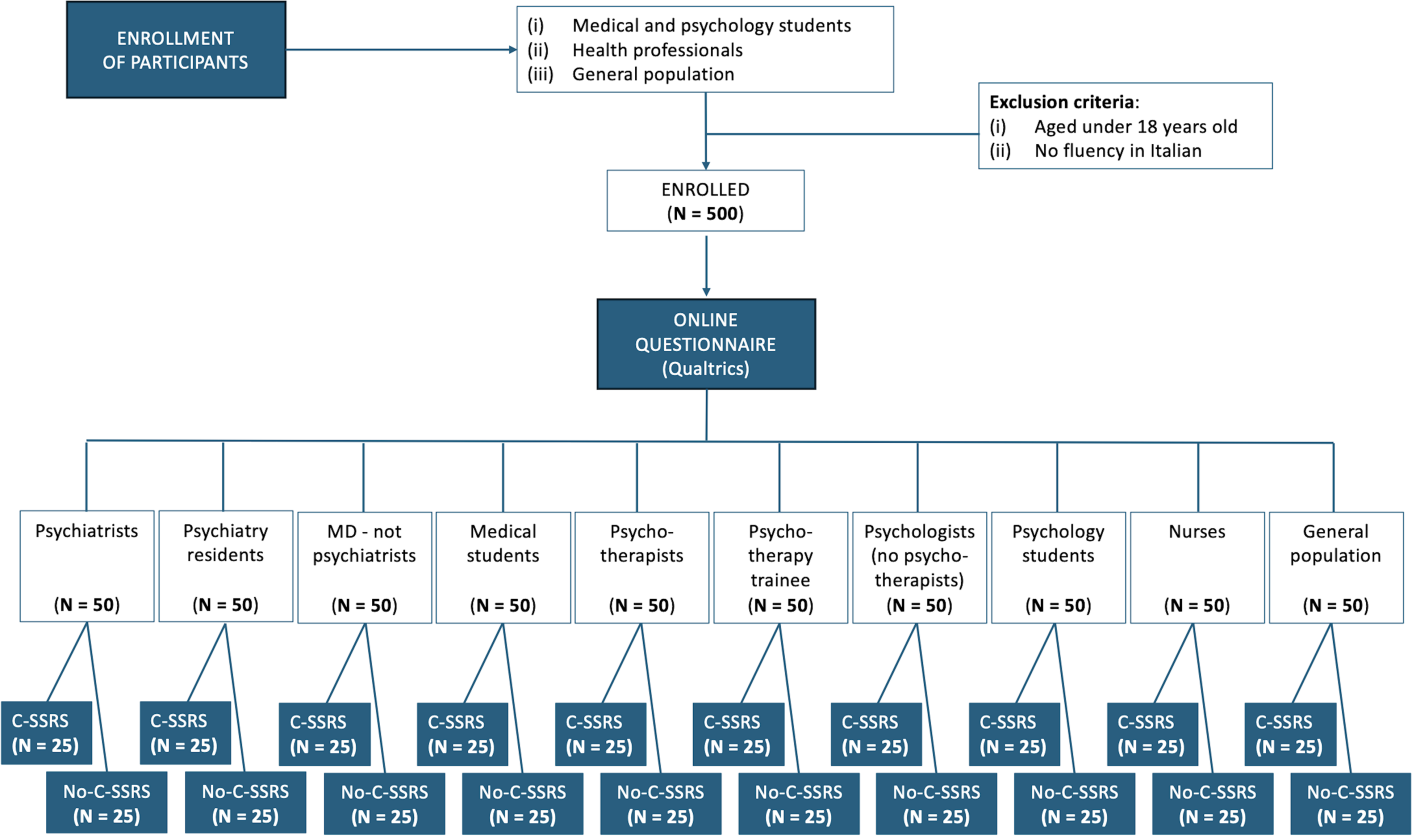
Flow-diagram showing procedures for enrollment in the study different groups of study participants.

The sample will include ten study groups, namely: (i) licensed psychiatrists, (ii) psychiatry residents, (iii) other medical doctors (non-psychiatrist), (iv) medical students, (v) licensed psychotherapists, (vi) psychotherapy trainees, (vii) psychologists (without psychotherapy training), (viii) psychology students, (ix) nurses, and (x) individuals from the general population without medical or psychological academic background (**Figure 1**). All participants belonging to one of the study groups who provide informed consent will be eligible to participate in the study, except those under 18 years of age and those with no fluency in the Italian language.

#### 2.2.2 Recruitment

Recruitment will take place exclusively via online diffusion of the link that will direct potential participants to the questionnaire. The link will be disseminated through official and non-official channels, including professional healthcare and student communities. Although recruitment is based on voluntary participation and therefore constitutes a non-probabilistic convenience sampling strategy, efforts will be made to enhance heterogeneity across professional roles and settings. Dissemination will target multiple institutions, professional associations, university programs, and healthcare services across different geographic areas. Invitations will be distributed through both academic and clinical networks to reach participants working in public hospitals, private practices, community mental health services, and primary care settings, as well as students enrolled in different universities. The study will be conducted as a remotely administered, vignette-based randomized design embedded within an online survey. The remote administration of this study will facilitate the broad dissemination of the questionnaire and enable the inclusion of participants affiliated with different institutions and geographic areas. Recruitment will last a total of 6 months, and participants will be free to complete the baseline questionnaire at any time during this period.

### 2.3 Procedure

#### 2.3.1 Baseline assessment

**Figure 2:**
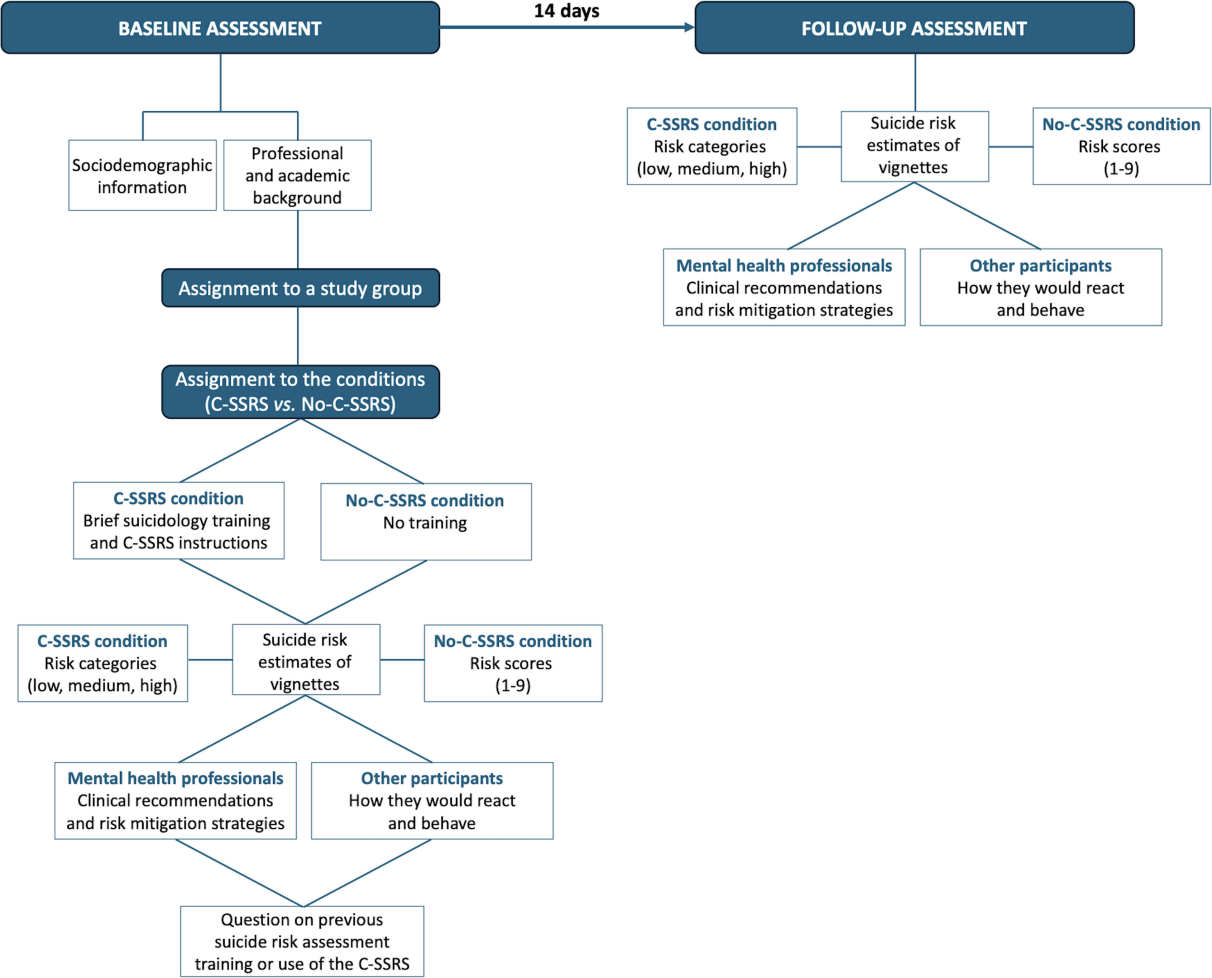
Conceptual framework of the CARE study showing the baseline and follow-up assessments.

The study procedures and enrollment process are shown in **Figure 2**. During the baseline assessment, participants will be asked to answer sociodemographic questions as well as to indicate their professional and academic background, in order to assign them to a specific study group. Then, participants of each group (i–x) will be randomly assigned to one of the two conditions (C-SSRS *vs.* No-C-SSRS).

The questionnaire will be designed so that each participant will be randomly assigned to 6 different vignettes to be evaluated, 2 for each risk category (low, moderate, and high). Specifically, participants assigned to the C-SSRS condition will complete a brief training module on basic suicidology notions embedded within the online questionnaire before evaluating the vignettes. The module is delivered as a single self-administered page and requires approximately 5 minutes to be completed. It is divided into two sections: first, it will provide concise definitions of suicide-related constructs, including thoughts of death, suicidal ideation, suicidal planning, non-suicidal self-injury, suicide attempt, and suicide death, in order to reduce interpretative variability across respondents; second, it will present step-by-step instructions on how to use the C-SSRS (14), explaining the logic of the six screening questions, when conditional questions are administered, and how responses map onto the three risk categories defined by the scale (low, moderate, high). Ultimately, participants will be provided with access to the official Italian version of the C-SSRS and informed that a mobile application version can be used if preferred. No feedback, supervision, or competency assessment will be provided, thereby reflecting the type of brief orientation often available in real-world implementation settings. The remaining participants will be asked to provide a subjective estimation of perceived suicide risk based on their impressions, knowledge, personal judgment, or clinical experience (for clinicians). Notably, for participants without a clinical background, the task is framed as an estimation of perceived suicide risk rather than a diagnostic or clinical evaluation. They will not be required to make clinical decisions but are instead asked to describe how they would interpret and respond to the situation described in the vignette. Participants assigned to the C-SSRS condition will be instructed, for each vignette, to provide a categorical rating of suicide risk as defined by the scale (low, moderate, high); whereas those assigned to the No-C-SSRS condition will be asked, for each vignette, to provide an ordinal risk estimate on a scale from 1 (very low risk) to 9 (imminent risk). Notably, the use of different response formats across conditions reflects the distinct decision-making frameworks under investigation. The C-SSRS operationalizes suicide risk through predefined categorical classifications and does not provide a continuous scoring system, while a 1–9 ordinal scoring metric will be adopted for the No-C-SSRS condition to better capture the natural variability of subjective risk appraisal without imposing a fixed, limited categorization. This approach allows the study to compare two ecologically valid assessment paradigms, structured categorical evaluation versus dimensional judgment, rather than two versions of the same measurement model.

In addition, licensed mental health professionals (psychiatrists and licensed psychologists) will be asked to provide clinical recommendations and to describe risk mitigation strategies they would use for each vignette scenario, whereas all other participants will be asked to describe how they would respond or behave in the described situations. At the end of the vignette assessment, all participants will be asked to report whether they have ever attended specialized training focused on suicide risk assessment or whether they have previously used the C-SSRS.

#### 2.3.2 Follow-up assessment

Two weeks after completing the baseline questionnaire, each participant will receive an email reminder to complete the follow-up questionnaire. The follow-up assessment will require participants to evaluate 6 vignettes (2 per risk category), randomly selected from the same pool of 18 vignettes, to examine consistency between baseline and follow-up ratings. Notably, different vignettes are intentionally administered at follow-up to avoid recall effects and to evaluate the temporal stability of participants’ general risk estimation approach rather than agreement on identical cases. Participants assigned to the C-SSRS condition at baseline will again be asked to provide a categorical rating of suicide risk as defined by the C-SSRS (low, moderate, high), although no informational training will be provided at follow-up. Concurrently, participants in the No-C-SSRS condition will be asked, for each vignette, to provide an ordinal suicide risk rating from 1 (very low risk) to 9 (imminent risk), following the same procedure used at baseline. If participants do not complete the follow-up questionnaire within five days of receiving the first reminder, up to three additional reminders will be sent, each one three to five days apart.

### 2.4 Variables and outcomes

#### 2.4.1 Study variables

The online questionnaire will collect sociodemographic data (e.g., age, sex, educational level) and professional and academic background information. Specifically, all participants will be asked to indicate whether they have a professional or academic background in the medical or psychological fields (e.g., professional role, current year of psychiatry residency, years since completion of psychiatry specialization, current year of psychotherapy training, years since completion of psychotherapy specialization, year of study in a psychology degree program, year of study in a medical degree program), their specific field of practice (e.g., medical specialty area, psychotherapy orientation, nursing field, no prior experience in the medical or psychological field), and their clinical experience (e.g., years of practice, primary psychotherapy orientation, supervision, years of personal therapy). Responses to these preliminary questions will be used to automatically assign each participant to the appropriate study group. After the completion of the suicide risk assessment based on six vignettes describing hypothetical scenarios, all participants will be asked to indicate whether they have ever completed a suicide risk assessment training or previously used the C-SSRS scale.

#### 2.4.2 Outcomes

The primary outcome will be the assessment of discrepancies in suicide risk estimates across study groups (between-subjects) and the evaluation of the stability of individual risk appraisal tendencies over time (within-subject), in order to identify specific patterns and factors that may influence variability and inter-rater agreement in risk estimation across different clinical scenarios.

The secondary outcome is the assessment of discrepancies in suicide risk estimates between the C-SSRS and No-C-SSRS conditions, in order to determine whether the use of a standardized suicide risk assessment instrument will lead to greater homogeneity in risk ratings across the study groups. To allow comparison of discrepancies between the two subgroups, suicide risk assessment will be expressed as categorical variables for participants assigned to the C-SSRS condition (low, moderate, high) and as ordinal scores for participants in the No-C-SSRS condition (from 1 to 9). Cut points were defined *a priori* to represent three clinically interpretable categories of risk intensity, corresponding to low (scores 1-3), intermediate (4–6), and high (7–9) perceived risk. This mirrors the three-level structure imposed by the C-SSRS while preserving the ordinal distribution of the original ratings (**Table 1**).

**Table 1:** Conversion of the suicide risk estimates between the C-SSRS and No-C-SSRS conditions.

C-SSRS condition risk categories	No-C-SSRS condition risk scores
Low risk	1-2–3 scores
Moderate risk	4-5–6 scores
High risk	7-8–9 scores

### 2.5 Data analysis plan

#### 2.5.1 Power analysis

The sample size calculation was conducted considering the primary between-subjects objective of the study, namely the comparison of suicide risk ratings across the ten participant groups at baseline. Although the study design includes repeated measurements within individuals, due to multiple vignette ratings and a follow-up assessment at two weeks, the power analysis was intentionally restricted to the primary baseline comparison in order to align with standard assumptions for sample size estimation, thus providing a conservative estimate of the required sample size.

The primary outcome for power estimation was operationalized as the vignette-based suicide risk rating expressed on a 1 to 9 scale in the No-C-SSRS condition, treated as an ordinal variable. Accordingly, the sample size calculation was based on a one-way ANOVA comparing the ten independent study groups. Although the 1 to 9 rating scale will be analyzed using ordinal mixed-effects models in the primary analyses, ANOVA will be adopted for sample size estimation as a conventional and conservative approximation for planning purposes when dealing with multi-level ordinal variables treated as approximately continuous. Assuming a small-to-medium effect size (f = 0.20), a two-sided alpha level of 0.05, and a desired statistical power of 80 percent (1 − β = 0.80), G*Power indicated that a minimum of 400 participants would be required to detect overall group differences. Although participants are randomized across two conditions (C-SSRS vs. No-C-SSRS), the primary power estimation focused on detecting between-group differences in suicide risk estimates at baseline. Randomization is expected to be approximately balanced across the ten groups, thereby preserving adequate sample size within each group for primary comparisons. This approach provides a conservative estimate of statistical power, as it does not incorporate the additional information obtained from repeated vignette ratings or from mixed-effects modeling, both of which are expected to increase precision and statistical efficiency.

To account for expected attrition at follow-up, the planned sample size was increased to at least 500 participants. This choice was informed by evidence from a recent study by Kolochowski et al. (20), which used the same set of vignettes adopted in the present protocol and reported a follow-up completion rate of 55.9 percent. Assuming a conservative attrition rate of approximately 40 to 45 percent, an initial sample of 500 participants would result in a final sample of approximately 275 to 300 individuals, which remains adequate for the planned longitudinal and within-subject analyses. The online administration format and broad inclusion criteria further support the feasibility of achieving this sample size.

#### 2.5.2 Planned statistical analysis

Analyses will examine discrepancies in suicide risk assessment across participant groups and over time to identify factors associated with variability. Descriptive statistics will summarize sociodemographic and professional characteristics of the sample, distribution of risk ratings, and follow-up completion rates. Given the hierarchical structure of the data, with multiple vignette ratings nested within participants, mixed-effects regression models will constitute the primary analytical approach. Random intercepts will be specified for participants to account for repeated ratings within individuals and, when appropriate, for vignettes to account for shared variance attributable to scenario characteristics. Fixed effects will include study group, experimental condition (C-SSRS vs. No-C-SSRS), time (baseline vs. follow-up), and their interactions.

Ratings collected on the 1 to 9 scale will be treated as ordinal variables. Accordingly, ordinal mixed-effects models will be used as the primary approach, whereas linear models treating the scale as continuous will be conducted only as sensitivity analyses to ensure robustness of findings. To evaluate potential bias related to categorization thresholds, sensitivity analyses will include alternative binning strategies (e.g., 1–2 vs. 3–7 vs. 8–9) and models using the full 1–9 scale without categorization.

To quantify inter-rater agreement across raters for each vignette, Krippendorff’s alpha will be used as the primary agreement index, as it is appropriate for multi-rater settings with potentially unbalanced data. Weighted kappa coefficients will be computed only in supplementary pairwise comparisons to facilitate interpretability relative to commonly reported agreement metrics. Within-person analyses will evaluate the stability of participants’ overall level and pattern of risk estimates across time using different vignette sets drawn from the same risk-calibrated pool. These analyses do not represent test–retest reliability of identical stimuli but rather the temporal consistency of risk perception. Because different vignettes of the same risk category will be administered at baseline and follow-up, within-person analyses will be conducted on participant-level summary indices (e.g., central tendency of ratings and distribution of risk categories across vignettes at each time point) rather than on vignette-level paired comparisons. For the No-C-SSRS condition, paired non-parametric tests, such as the Wilcoxon signed-rank test, and correlation analyses will be used. For the C-SSRS condition, indices of temporal consistency will be computed to evaluate the stability of participants’ overall tendency to classify scenarios into risk categories across time, rather than agreement on identical stimuli.

The mixed-effects framework described above will also be used to examine interaction effects. Pre-specified interaction terms will examine whether differences in risk estimation vary as a function of demographic and professional variables (e.g., age, gender, years of clinical experience, prior suicide-specific training) across study group and experimental condition. In addition, to control for inflation of Type I error due to multiple comparisons, p-values from secondary analyses will be adjusted using the false discovery rate approach.

Missing data will be handled under a missing-at-random assumption using maximum likelihood estimation within mixed-effects models, which allows inclusion of all available observations without listwise deletion. Attrition between baseline and follow-up will be examined descriptively, and sensitivity analyses will compare participants who completed follow-up with those who did not. Predictors of attrition will be explored to assess potential systematic differences between completers and non-completers.

### 2.6 Data access and management

Data will be managed and stored within the Department of Pathophysiology and Transplantation at the University of Milan and will be accessed exclusively by the study investigators and will not be shared with other operational units. All collected data, including personal data, will be the exclusive property of the University of Milan, and no external parties will have access to them. The questionnaire will be administered through a dedicated link redirecting participants to the Qualtrics platform. Responses will be collected directly through Qualtrics and subsequently organized into a study-specific database. Participation in the baseline questionnaire will be completely anonymous. At the end of the baseline survey, participants will be invited, on a fully voluntary basis, to provide an email address exclusively for the purpose of receiving the follow-up questionnaire link. Providing an email address will not be mandatory for participation in the study, and individuals may complete the baseline survey without leaving any contact information. Contact information will be used solely for follow-up communication and will not be included in the analytical dataset. Following data extraction, study variables and suicide risk estimates results will be entered into an electronic database for statistical analysis. In accordance with applicable data protection regulations (General Data Protection Regulation, EU Regulation No. 2016/679), all collected data will be stored confidentially for a period of ten years within the Department of Pathophysiology and Transplantation at the University of Milan.

### 2.7 Ethics and regulatory approval

The study protocol of the CARE project was reviewed and approved by the ethics committee of the University of Milan (Ref: 91/25), and all subjects will provide electronic informed consent in accordance with the Declaration of Helsinki.

## 3 Discussion

This study protocol outlines an innovative research project designed to examine both between-person and within-person variability in suicide risk estimates across several groups, including mental health professionals, other health professionals, medical and psychology students, and individuals from the general population. The study aims to produce evidence that can support enhancements in the training of healthcare professionals and the development of more consistent and reliable risk assessment strategies. Rather than attempting to improve statistical prediction of suicidal behavior, the CARE study focuses on the variability of human judgment in suicide-related situations, an issue that remains highly relevant even in clinical models that prioritize individualized formulation over categorical risk stratification. Furthermore, the study evaluates the effect of a brief, scalable training on a standardized instrument, the C-SSRS, reflecting real-world conditions in which such tools are frequently introduced without extensive training. Therefore, a transversal objective of this study is to determine whether the use of a standardized instrument, the C-SSRS, may reduce subjectivity in judgment and promote greater uniformity in suicide risk estimates across clinical and non-clinical participants. Indeed, understanding how non-professionals estimate suicide risk is important because individuals frequently encounter situations involving psychological distress in family, social, or workplace contexts, where interpretation of warning signs may influence help-seeking or referral behaviors. Additionally, by presenting different yet comparable vignettes at follow-up, the study examines whether individuals display stable risk attribution patterns across contexts, an approach that more closely reflects real-world clinical decision-making where identical cases are never reencountered.

Suicide risk assessment is challenging and often inaccurate, even among experienced clinicians, particularly when it relies on subjective risk categorization (21). Furthermore, studies have shown that the use of risk scales or psychosocial assessment protocols has not significantly improved the reliability of suicide prediction (28–30). This issue can be attributed, at least in part, to insufficient training in suicide risk assessment among healthcare professionals, who often exhibit an ambivalent attitude toward suicide, which substantially affects prevention efforts and clinical decision-making (31). Additionally, many psychologists tend to avoid treating patients at high suicide risk (32), and most healthcare professionals lack suicide specific knowledge, often without having received any training in suicide prevention or assessment (33). This limitation undermines their ability to recognize warning signs and effectively identify individuals at greatest risk (34, 35). For example, in a study of 670 healthcare professionals, among those who had experienced the suicide loss of a patient, 44% reported that they had not received adequate professional training on suicide-related knowledge (36). Conversely, completing suicidology training increases confidence and improves the management of suicide risk, even among early career psychiatrists and trainees (37). In the general population, although knowledge about suicide can be lifesaving across all contexts, it is often extremely limited and consistently shaped by misconceptions, prejudices, misinformation, and stigma across different cultures (38–40).

Given these considerations, the expected findings of the CARE project have the potential to yield meaningful insights regarding the impact of brief suicidology training and the use of a standardized tool for suicide risk assessment, the C-SSRS, among healthcare professionals and the general population. Furthermore, identifying the study groups that exhibit greater variability in suicide risk estimates, along with the academic and professional factors associated with this variability, may inform the development of targeted psychoeducational interventions aimed at improving the accuracy and consistency of suicide risk estimates. The results of this project may also guide refinements in professional training for psychiatrists, psychologists, nurses, and other healthcare providers by promoting the integration of standardized assessment practices and structured decision-making. However, recruitment through online dissemination constitutes a convenience sampling strategy and may limit representativeness. Participants may differ from non-participants in terms of interest in suicidology, motivation, or prior exposure to suicide-related content. Although efforts will be made to recruit across diverse healthcare settings and institutions, findings should be interpreted with caution regarding generalizability to all clinicians or to the broader general population. The primary objective of the study is comparative, focusing on relative differences in risk appraisal patterns across groups rather than population-level estimates. In addition, because participation in the follow-up assessment requires participants to voluntarily provide contact information (e-mail) after completing an otherwise fully anonymous baseline survey, a self-selection bias may be introduced, whereby individuals who agree to be recontacted could differ systematically from those who choose to remain anonymous. Ultimately, comparing healthcare professionals and the general population may offer valuable insights for planning awareness campaigns and preventive interventions that extend beyond clinical settings, including educational, community-based, and public health environments. A deeper understanding of how laypeople interpret warning signs and estimate suicide risk, indeed, may support the development of more effective communication strategies, thereby improving early recognition of risk and facilitating timely referral to appropriate services.

Overall, this project will represent one of the largest and most comprehensive initiatives aimed at comparing suicide risk assessment among mental health professionals, other healthcare professionals, and the general population. It will also provide insight into the effects of using standardized tools such as the C-SSRS on inter-rater and intra-rater variability, ultimately generating knowledge that can meaningfully contribute to strengthening suicide prevention efforts.
